# Trigonometrically-fitted second derivative method for oscillatory problems

**DOI:** 10.1186/2193-1801-3-304

**Published:** 2014-06-24

**Authors:** Fidele Fouogang Ngwane, Samuel Nemsefor Jator

**Affiliations:** Department of Mathematics, USC Salkehatchie, Allendale, SC 29810 USA; Department of Mathematics and Statistics, Austin Peay State University, Clarksville, TN 37044 USA

**Keywords:** Oscillatory initial value problems, Trigonometrically-fitted second derivative method, Stability

## Abstract

**Abstract:**

A continuous Trigonometrically-fitted Second Derivative Method (CTSDM) whose coefficients depend on the frequency and stepsize is constructed using trigonometric basis functions. A discrete Trigonometrically-fitted second derivative method (TSDM) is recovered from the CTSDM as a by-product and applied to solve initial value problems (IVPs) with oscillating solutions. We discuss the stability properties of the TSDM and present numerical experiments to demonstrate the efficiency of the method.

**AMS Subject Classification:**

65L05; 65L06

## Introduction

In this paper, we consider the subclass of first order differential equation
1

with periodic or oscillating solutions where *f*:ℜ×ℜ^*m*^→ℜ^*m*^, *y*,*y*_0_∈ℜ^*m*^. Oscillatory IVPs frequently arise in areas such as classical mechanics, celestial mechanics, quantum mechanics, and biological sciences. Several numerical methods based on the use of polynomial basis functions have been developed for solving this class of important problems (see Lambert 1991, 1973, Hairer et al. in (Hairer and Wanner 1996), Hairer 1982, and Sommeijer 1993). Other methods based on exponential fitting techniques which take advantage of the special properties of the solution that may be known in advance have been proposed (see Simos 1998, Vanden Berghe et al. 2001a, Vanden Berghe et al. 2009, Vigo-Aguiar et al. 2003, Franco 2002, Fang et al. 2009, Nguyen et al. 2007, Ozawa 2005, Jator et al. 2012, and Ngwane et al. 2012b). In the spirit of 2005, the motivation governing the exponentially-fitted methods is inherent to the fact that if the frequency or a reasonable estimate of it is known in advance, these methods will be more advantageous than the polynomial based methods.

The aim of this paper is to construct a TSDM. This construction is done by initially developing a CTSDM which then provides a discrete method that is applied as a TSDM which takes the frequency of the solution as a priori knowledge. In particular, CTSDM consists of a sum of continuous functions while TSDM is a by-product of CTSDM. The coefficients of the TSDM are functions of the frequency and the stepsize, hence the solutions provided by the proposed method are highly accurate if (1) has periodic solutions with known frequencies. We adopt the approach given in Jator et al. in (Ngwane and Jator 2012a; Jator et al. 2012), where the TSDM is used to obtain the approximation *y*_*n*+1_ to the exact solution *y*(*x*_*n*+1_) on the interval [ *x*_*n*_,*x*_*n*+1_], *h*=*x*_*n*+1_−*x*_*n*_, *n*=0,…,*N*−1, on a partition [ *a*,*b*], where , *h* is the constant stepsize, *n* is a grid index and *N*>0 is the number of steps. We note that second derivative methods with polynomial basis functions were proposed to overcome the Dahlquist 1956 barrier theorem whereby the conventional linear multistep method was modified by incorporating the second derivative term in the derivation process in order to increase the order of the method, while preserving good stability properties (see Enright 1974).

This paper is organized as follows. In Section “’’, we obtain a trigonometric basis representation *U*(*x*) for the exact solution *y*(*x*) which is used to generate a TSDM for solving (1). The analysis and implementation of the TSDM are discussed in Section “Error analysis and stability” to show the accuracy and efficiency of the TSDM. Finally, we give some concluding remarks in Section “Conclusion”.

## Development of method

In this section, our objective is to construct a CTSDM which produces a discrete method as a by-product. The method has the form
2

where *u*=*wh*, *β*_*j*_(*u*), *γ*_*j*_(*u*), *j*=0,1, are coefficients that depend on the stepsize and frequency. We note that *y*_*n*+*j*_ is the numerical approximation to the analytical solution *y*(*x*_*n*+*j*_), and


with *j*=0,1. In order to obtain equation (2) we proceed by seeking to approximate the exact solution *y*(*x*) on the interval [ *x*_*n*_,*x*_*n*_+*h*] by the interpolating function *U*(*x*) of the form
3

where *a*_0_,*a*_1_,*a*_2_,*a*_3_ and *a*_4_ are coefficients that must be uniquely determined. We then impose that the interpolating function in (3) coincides with the analytical solution at the point *x*_*n*_ to obtain the equation
4

We also demand that the function (3) satisfies the differential equation (1) at the points *x*_*n*+*j*_, *j*=0,1 to obtain the following set of three equations:
5

Equations (4) and (5) lead to a system of five equations which is solved by Cramer’s rule to obtain *a*_*j*_, *j*=0,1,2,3,4. Our continuous CTSDM is constructed by substituting the values of *a*_*j*_ into equation (3). After some algebraic manipulation, the CTSDM is expressed in the form
6

where *w* is the frequency, *β*_0_(*w*,*x*), *β*_1_(*w*,*x*), *γ*_0_(*w*,*x*), and *γ*_1_(*w*,*x*), are continuous coefficients. The continuous method (6) is used to generate the method of the form (2). Thus, evaluating (6) at *x*=*x*_*n*+1_ and letting *u*=*wh*, we obtain the coefficients of (2) as follows:
7

## Error analysis and stability

### Local truncation error

We note that when *u*→0 the coefficients given by (7) are vulnerable to heavy cancellations and hence the following Taylor series expansion must be used (see Simos 1998).
8

In fact, for practical computations when *u* is small, it is better to use the series expansion (8) (see Calvo et al. 2009).

Thus the Local Truncation Error (LTE) of (2) subject to (8) is obtained as
9

#### **Remark1**

The method (2) specified by (8) is a fourth-order method and reduces to a one-step conventional second derivative method as *u*→0 (see Lambert 1973, p. 201).

### Stability

#### **Proposition1**

The TSDM (2) applied to the test equations *y*^′^=*λy* and *y*^′′^=*λ*^2^*y* yields
10

with
11

#### Proof

We begin by applying (2) to the test equations *y*^′^=*λy* and *y*^′′^=*λ*^2^*y* which are expressed as *f*(*x*,*y*)=*λy* and *g*(*x*,*y*)=*λ*^2^*y* respectively; letting *q*=*hλ* and *u*=*wh*, we obtain a linear equation which is used to solve for *y*_*n*+1_ with (11) as a consequence.

#### **Remark2**.

The rational function *M*(*q*;*u*) is called the stability function which determines the stability of the method.

#### **Definition1**

A region of stability is a region in the *q*−*u* plane, in which |*M*(*q*;*u*)|≤1.

The TSDM method (2) specified by (7) is given by
12

#### **Definition2**.

The method (12) is zero-stable provided the roots of the first characteristic polynomial have modulus less than or equal to unity and those of modulus unity are simple (see Lambert 1991).

#### **Definition3**.

The method (12) is consistent if it has order *p*>1 (see (Fatunla 1991)).

#### **Remark3**.

The TSDM (12) is consistent as it has order *p*>1 and zero-stable, hence it is convergent since zero-stability + consistency = convergence.

#### **Corollary1**

The TSDM (12) has *M*(*q*;*u*) specified by


#### **Remark4**.

In the *q*−*u* plane the TSDM (12) is stable for *q*≤0, and *u*∈[ −2*π*,2*π*], since from above |*M*(*q*;*u*)|≤1, *q*≤0.

#### **Remark5**.

**Figure 1:**
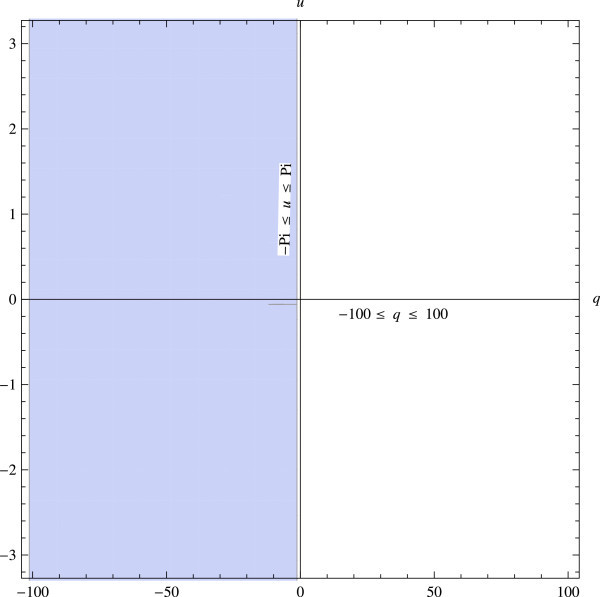
**The shaded region represents the truncated region of absolute stability.**

**Figure 2:**
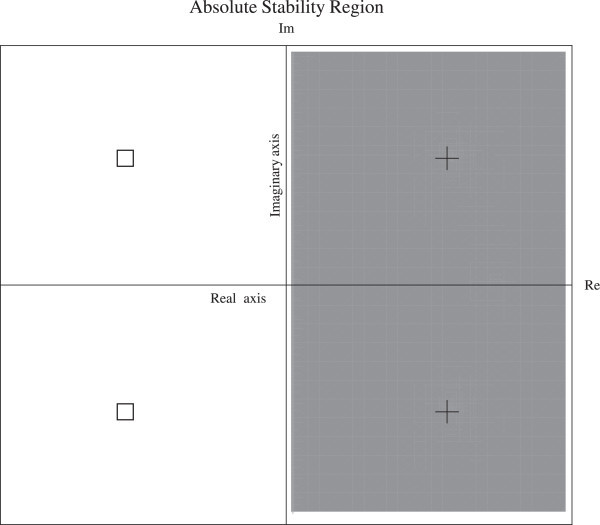
***M*** **(** ***q*** **;** ***u*** **) has zeros**  **and no poles** ***(+)*** **in**  **, with** ***u*** **=** ***π*** **.**

Figure 1 is a plot of the stability region and Figure 2 shows the zeros and poles of *M*(*q*;*u*). We note from Figure 2 that the stability region includes the entire left side of the complex plane.

#### **Definition4**.

The TSDM with the stability function (11) is said to be A-stable at *u*=*u*_0_, −2*π*≤*u*_0_≤2*π*, if |*M*(*q*;*u*)|≤1, , (see Nguyen et al. 2007).

#### **Remark6**.

We observe from definition 1, remarks 4, 5, and Figure 2, that TSDM is *A*-stable. In particular, |*M*(*q*;*iy*)|=1, and by the maximum principle, the method will be A-stable if |*M*(*q*;*u*)| has no poles in the left plane (see E. Hairer et al. 1996, p.43, 53). Moreover, the real part of the zeros of |*M*(*q*;*u*)| must be negative, while the real part of the poles of |*M*(*q*;*u*)| must be positive.

### Implementation

In the spirit of Ngwane et al. in (Ngwane and Jator 2012a; 2012b), the TSDM (12) is implemented to solve (1) without requiring starting values and predictors. For instance, if we let *n*=0 in (12), then *y*_1_ is obtained on the sub-interval [ *x*_0_,*x*_1_], as *y*_0_ is known from the IVP. Similarly, if *n*=1, then *y*_2_ is obtained on the sub-interval [ *x*_1_,*x*_2_], as *y*_1_ is known from the previous computation, and so on; until we reach the final sub-interval [ *x*_*N*−1_,*x*_*N*_]. We note that for linear problems, we solve (1) directly using the feature *solve*[ ] in Matlab, while nonlinear problems use the Newton’s method in Matlab enhanced by the feature *fsolve*[ ].

## Numerical examples

In this section, we give numerical examples to illustrate the accuracy (small errors) and efficiency (fewer number of function evaluations (NFEs)) of the TSDM. We find the approximate solution on the partition *π*_*N*_, where *π*_*N*_:*a*=*x*_0_<*x*_1_<*x*_2_<...<*x*_*n*_<*x*_*n*+1_<…<*x*_*N*_=*b*, and we give the errors at the endpoints calculated as Error= *y*_*N*_−*y*(*x*_*N*_). We note that the method requires only two function evaluations per step and in general requires (2*N*+2) NFEs on the entire interval. All computations were carried out using a written code in Matlab.

### **Example1**

Consider the given two-body problem which was solved by Ozawa 2005.


where *e*, 0≤*e*<1 is an eccentricity. The exact solution of this problem is


where *k* is the solution of the Kepler’s equation *k*=*x*+*e* sin(*k*). We choose *ω*=1.

Table 1 contains the results obtained using the TSDM. These results are compared with the explicit singly diagonally implicit Runge-Kutta (ESDIRK) and the functionally fitted ESDIRK (FESDIRK) methods given in Ozawa 2005. In terms of accuracy, Table 1 clearly shows that TSDM performs better than those in Ozawa 2005.

**Table 1:** **Results with** ***ω*** **=1,** ***e*** **=0** ***.*** **005, for Example 1**

TSDM		FESDIRK4(3)		ESDIRK4(3)	
***N***	| ***E*** ***r*** ***r*** ***o*** ***r***|	***N***	| ***E*** ***r*** ***r*** ***o*** ***r***|	***N***	| ***E*** ***r*** ***r*** ***o*** ***r***|
150	1.203×10^−2^	170	2.866×10^−1^	277	2.153×10^0^
200	5.694×10^−3^	225	7.846×10^−3^	496	1.494×10^−1^
300	3.143×10^−4^	381	1.399×10^−3^	884	9.359×10^−3^
600	1.259×10^−6^	680	1.690×10^−4^	1573	6.200×10^−4^
800	1.264×10^−7^	1207	1.846×10^−5^	2796	4.416×10^−5^
1600	4.947×10^−10^	2144	1.938×10^−6^	4970	3.412×10^−6^
2400	1.931×10^−11^	3806	1.993×10^−7^	8833	2.848×10^−7^
3200	1.944×10^−12^	6762	2.021×10^−8^	15706	2.530×10^−8^

### **Example2**.

We consider the nonlinear Duffing equation which was also solved Ixaru et al. 2004.


The analytic solution is given by


where *Ω*=1.01, *B*=0.002, *C*_0_=0.200426728069, *C*_1_=0.200179477536, *C*_2_=0.246946143×10^−3^, *C*_3_=0.304016×10^−6^, *C*_4_=0.374×10^−9^. We choose *ω*=1.01 and for more on frequency choice see Ramos et al. 2010.

We compare the end-point global errors for TSDM with the fourth order methods in Ixaru et al. 2004. We see from Table 2 that the results produced by TSDM are better than Simos’ method used in (Ixaru and Berghe 2004), as TSDM produces better error magnitude while using less number of steps and fewer number of function evaluations. TSDM is very competitive to the method used by Ixaru et al. 2004.

**Table 2:** **Results with** ***ω*** **=1** ***.*** **01, for Example 2**

TSDM		Simos		Ixaru et al.	
***N***	| ***E*** ***r*** ***r*** ***o*** ***r***|	***N***	| ***E*** ***r*** ***r*** ***o*** ***r***|	***N***	| ***E*** ***r*** ***r*** ***o*** ***r***|
150	3.3×10^−3^	300	1.7×10^−3^	300	1.1×10^−3^
300	6.4×10^−5^	600	1.9×10^−4^	600	5.4×10^−5^
600	5.1×10^−6^	1200	1.4×10^−5^	1200	1.9×10^−6^
2000	1.0×10^−7^	2400	8.7×10^−7^	2400	6.2×10^−8^

### **Example3**.

We consider the following inhomogeneous IVP by Simos 1998.


where the analytic solution is given by


The exponentially-fitted method in Simos 1998 is fourth order and hence comparable to our method, TSDM. We see from Table 3 that TSDM is more efficient than the method in Simos 1998. We also compare the computational efficiency of the two methods by considering the NFEs over *N* integration steps for each method. Our method, TSDM, requires only 2*N*+2 function evaluations in *N* steps compared to 4*N* function evaluations in *N* steps for the method in Simos 1998. Hence for this example, TSDM performs better.

**Table 3:** **Results with** ***ω*** **=10, for Example 3**

	TSDM		Simos 1998	
N	| ***E*** ***r*** ***r*** ***o*** ***r***|	NFEs	| ***E*** ***r*** ***r*** ***o*** ***r***|	NFEs
1000	1.7×10^−3^	4004	1.4×10^−1^	8000
2000	2.5×10^−4^	8004	3.5×10^−2^	16000
4000	2.7×10^−5^	16004	1.1×10^−3^	32000
8000	1.6×10^−6^	32004	8.4×10^−5^	64000
16000	1.0×10^−7^	64004	5.5×10^−6^	128000
32000	6.3×10^−9^	128004	3.5×10^−7^	256000

### **Example4**.

*Linear Kramarz problem We consider the following second-order IVP, (see Nguyen et al.*^2007^*[p. 204])*

We use this example to show the efficiency of TSDM on linear systems. Nguyen et al.^2007^ used the “trigonometric implicit Runge-Kutta”, TIRK3, method to solve the above linear Kramarz problem. Clearly, TSDM performs better as seen in Table 4.

**Table 4:** **Results with** ***ω=1*** **, for Example 4**

TSDM			Nguyen et al. 2007		
N	| ***E*** ***r*** ***r*** ***o*** ***r***|	NFEs	N	| ***E*** ***r*** ***r*** ***o*** ***r***|	NFEs
10	1.3×10^−15^	88	73	3.3×10^−12^	327
43	8.4×10^−14^	368	142	0.9×10^−11^	707
80	7.1×10^−15^	648	170	3.7×10^−12^	811

### **Example5**.

*We consider the IVP (see Vigo-Aguiar et al.*^2003^*)*

where *K*=314.16, and we choose *ω*=314.16. The analytic solution is given by


This problem demonstrates the performance of TSDM on a well-known oscillatory problem. We compare the results from TSDM with the Dissipative Chebyshev exponential-fitted methods, CHEBY24 and CHEBY1 used in Vigo-Aguiar et al.^2003^. We see that TSDM uses fewer number of function evaluations with better accuracy than CHEBY24 that is designed to use fewer number of steps. Integrating in the interval [ 0,1] with a stepsize equal to the total length of the interval, we obtain an error of order 10^−21^. Hence TSDM is a more efficient integrator. We note that compared with the methods CHEBY24 and CHEBY1 which use stepsizes considerably larger than those used in multistep methods, TSDM is very competitive and superior to both CHEBY24 and CHEBY1.

### **Example6**.

A nearly sinusoidal problem

We consider the following IVP on the range 0≤*t*≤10, (see Nguyen et al.^2007^, p. 205)


We choose *β*=−3 and *β*=−1000 in order to illustrate the phenomenon of stiffness. Given the initial conditions *y*_1_(0)=2 and *y*_2_(0)=3, the exact solution is *β*-independent and is given by


This example is chosen to demonstrate the performance of TSDM on stiff problems. We compute the solutions to Example (6) with *β*=−3, −1000. We obtain better absolute errors than Nguyen et al. (2007). This efficiency is achieved using fewer number of steps and less number of function evaluations than Nguyen et al. (2007). For example when *β*=−3, our method generates a solution with error magnitude 10^−6^ involving just 6 steps and 28 function evaluations, whereas (Nguyen et al. 2007) attains the same error magnitude using 10 steps and 47 function evaluations. When *β*=−1000, TSDM generates solutions with comparable error magnitude. We see that TSDM is competitive and better than the method in Nguyen et al. (2007) which is of order six and is thus expected to do better.

### An implementation in predictor-corrector mode

In this section, we also implement our CTSDM in a predictor-corrector mode. The predictor is given by
13

where
14

and the corrector is given by equations (6) and (7). We note that when *u*→0 we use the following Taylor series expansion (see Simos^1998^)
15

As we expected, the predictor-corrector (PreCor) mode runs faster than the TSDM but is less accurate compared to the TSDM. We illustrate this by applying the predictor-corrector to Example 2 and Example 3. We plot the efficiency curves showing the accuracy versus the CPU computation time, and the accuracy versus the NFEs.

### Estimating the frequency

A preliminary testing indicates that a good estimate of the frequency can be obtained by demanding that *LTE*=0, and solving for the frequency. That is, solve for *ω* given that  where *y*^(*j*)^,*j*=2,…,5 denote derivatives. We used this procedure to calculate *ω* for the problem given in example (5) and obtained *ω*≈± 314.16, which approximately gives the known frequency *ω*=314.16. Hence, this procedure is interesting and will be seriously considered in our future research.

We note that estimating the frequency and the choice of the frequency in trigonometrically-fitted methods is challenging and has grown in interest. Existing references on how to estimate the frequency and on the choice of the frequency include G. Vanden Berghe et al.2001b, and Ramos et al.^2010^.

## Conclusion

**Figure 3:**
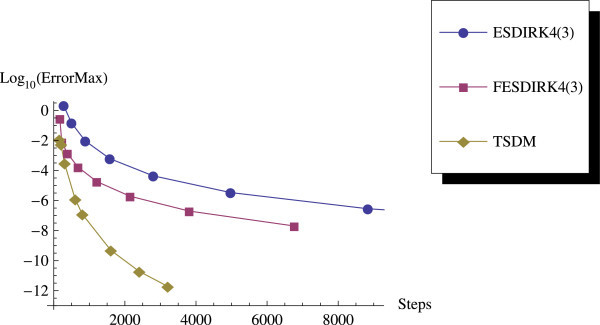
**Efficiency curves for Example 1.**

**Figure 4:**
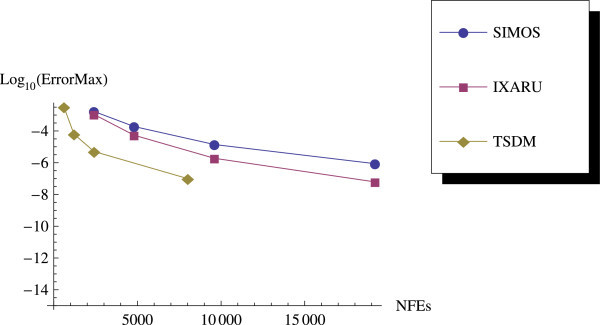
**Efficiency curves for Example 2.**

**Figure 5:**
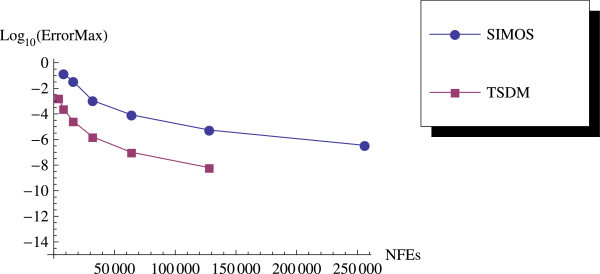
**Efficiency curves for Example 3.**

**Figure 6:**
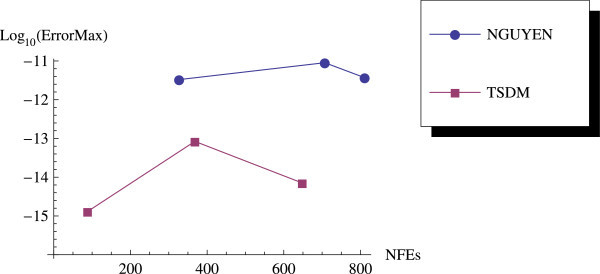
**Efficiency curves for Example 4.**

**Figure 7:**
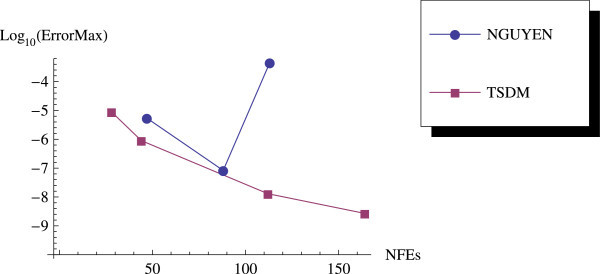
**Efficiency curves for Example 6 with** ***β*** **=−3.**

**Figure 8:**
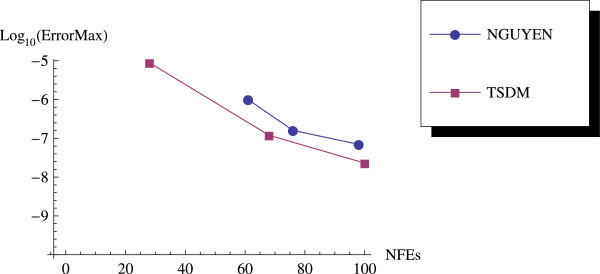
**Efficiency curves for Example 6 with** ***β*** **=−1000.**

**Figure 9:**
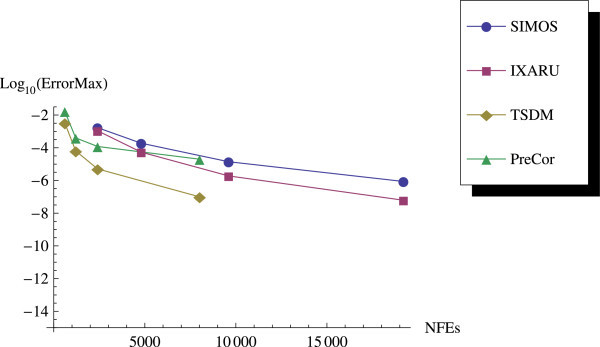
**Efficiency curve for Example 2 with predictor-corrector.**

**Figure 10:**
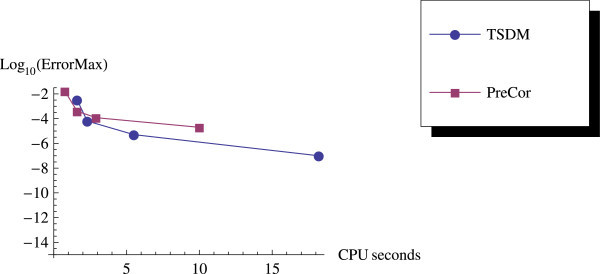
**Time efficiency curve for Example 2 with predictor-corrector.**

**Figure 11:**
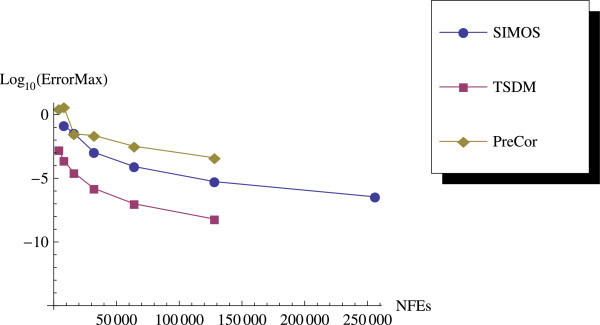
**Efficiency curve for Example 3 with predictor-corrector.**

**Figure 12:**
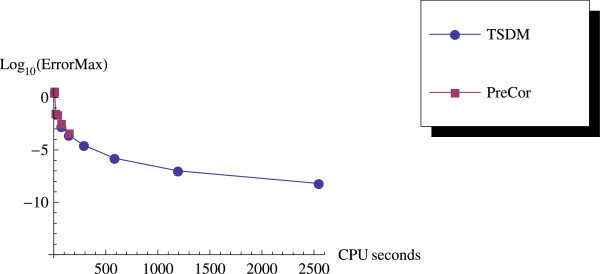
**Time efficiency curve for Example 3 with predictor-corrector.**

We have proposed a TSDM for solving oscillatory IVPs. The TSDM is *A*-stable and hence, an excellent candidate for solving stiff IVPs. This method has the advantages of being self-starting, having good accuracy with order 4, and requiring only two functions evaluation at each integration step. We have presented representative numerical examples that are linear, non-linear, stiff and highly oscillatory. These examples show that the TSDM is more accurate and efficient than those in Nguyen et al.^2007^, Simos^1998^, Ixaru et al.^2004^, and Ozawa^2005^. Details of the numerical results are displayed in Tables 1, 2, 3, 4, 5, 6, 7, 8, 9 and 10 and the efficiency curves are presented in Figures 3, 4, 5, 6, 7, 8, 9, 10, 11 and 12. Our future research will incorporate a technique for accurately estimating the frequency as suggested in subsection “Estimating the frequency” as well as implementing the method in a variable step mode.

**Table 5:** **Results with** ***ω*** **=314** ***.*** **16, for Example 5 on [ 0,100]**

TSDM			CHEBY24		
N	| ***E*** ***r*** ***r*** ***o*** ***r***|	NFEs	N	| ***E*** ***r*** ***r*** ***o*** ***r***|	NFEs
9	5.9×10^−14^	40	9	1.84×10^−11^	450
20	4.0×10^−15^	84	-	-	-

**Table 6:** **Results with** ***ω*** **=314** ***.*** **16, for Example 5 on [ 0,1]**

TSDM			CHEBY1		
N	| ***E*** ***r*** ***r*** ***o*** ***r***|	NFEs	N	| ***E*** ***r*** ***r*** ***o*** ***r***|	NFEs
1	1.29×10^−21^	8	1	1×10^−16^	8

**Table 7:** **Results with** ***ω*** **=1, for Example 6 with** ***β*** **=−3**

TSDM with( ***β***=−3)			Nguyen et al. 2007 with ( ***β***=−3)		
N	| ***E*** ***r*** ***r*** ***o*** ***r***|	NFEs	N	| ***E*** ***r*** ***r*** ***o*** ***r***|	NFEs
6	8.9×10^−6^	28	10	5.4×10^−6^	47
10	9.0×10^−7^	44	19	8.3×10^−8^	88
27	1.3×10^−8^	112	23	4.5×10^−4^	113
40	2.7×10^−9^	164	-	−	-

**Table 8:** **Results with** ***ω*** **=1, for Example 6 with** ***β*** **=−1000**

TSDM with ( ***β***=−1000)			Nguyen et al. 2007 with ( ***β***=−1000)		
N	| ***E*** ***r*** ***r*** ***o*** ***r***|	NFEs	N	| ***E*** ***r*** ***r*** ***o*** ***r***|	NFEs
6	8.9×10^−6^	28	13	1.0×10^−6^	61
16	1.2×10^−7^	68	16	1.6×10^−7^	76
24	2.3×10^−8^	100	21	7.0×10^−8^	98

**Table 9:** **Results, with predictor-corrector (PreCor) and** ***ω*** **=1** ***.*** **01, for Example 2**

	TSDM		PreCor		Simos		Ixaru et al.	
***N***	| ***E*** ***r*** ***r*** ***o*** ***r***|	CPU	| ***E*** ***r*** ***r*** ***o*** ***r***|	CPU	***N***	| ***E*** ***r*** ***r*** ***o*** ***r***|	***N***	| ***E*** ***r*** ***r*** ***o*** ***r***|
150	3.3(−3)	1.6	1.7(−2)	0.76	300	1.7(−3)	300	1.1(−3)
300	6.4(−5)	2.3	4.0(−4)	1.6	600	1.9(−4)	600	5.4(−5)
600	5.1(−6)	5.5	1.2(−4)	2.9	1200	1.4(−5)	1200	1.9(−6)
2000	1.0(−7)	18.2	2.0(−5)	10	2400	8.7(−7)	2400	6.2(−8)

**Table 10:** **Results, with predictor-corrector (PreCor) and** ***ω*** **=1, for Example 3**

	TSDM			PreCor		Simos 1998	
N	NFEs	CPU	| ***E*** ***r*** ***r*** ***o*** ***r***|	CPU	| ***E*** ***r*** ***r*** ***o*** ***r***|	NFEs	| ***E*** ***r*** ***r*** ***o*** ***r***|
1000	4004	73	1.7(−3)	4.8	2.9(0)	8000	1.4(−1)
2000	8004	145	2.5(−4)	9.5	4.1(0)	16000	3.5(−2)
4000	16004	290	2.7(−5)	19	3.1(−2)	32000	1.1(−3)
8000	32004	584	1.6(−6)	38	2.3(−2)	64000	8.4(−5)
16000	64004	1194	1.0(−7)	75	3.3(−3)	128000	5.5(−6)
32000	128004	2546	6.3(−9)	150	4.1(−4)	256000	3.5(−7)
